# Determinants of international Korean language promotion: A cross-country analysis

**DOI:** 10.1016/j.heliyon.2023.e21078

**Published:** 2023-10-16

**Authors:** Xingong Ding, Yujiao Wu

**Affiliations:** a Department of International Trade, Jeonbuk National University, South Korea; b School of Asian Studies, Beijing Foreign Studies University, China

**Keywords:** TOPIK, Hallyu, Motivation for learning Korean, Heckman two-step method, International promotion of language

## Abstract

This study investigates the determinants of the international promotion of the Korean language at the cross-country level. The analysis is based on candidate data drawn from the Test of Proficiency in Korean (TOPIK) and uses a cross-country panel data model to measure how several factors affect Korea's international promotion. To mitigate sample selection bias, we estimated the model using the Heckman two-step method. The findings show that factors such as the Hallyu (or “Korean Wave”), international business, motivation to migrate to South Korea, motivation to study in South Korea, and Korean language learning resources play essential roles in the international promotion of Korean. The development level of the sample countries influenced the importance of each factor. For example, Hallyu, often regarded as important for the international promotion of Korean, was shown to be statistically significant only in the sample of developed countries. In addition, a shortage of Korean language learning resources has been identified as a major impediment to the promotion of Koreans in developing countries. These results provide new insights that can assist in formulating policies to promote the Korean language worldwide.

## 1 Introduction

Korean has been promoted worldwide over the past decade as the global demand for the language has surged. Approximately 2000 institutions outside South Korea offer Korean-language courses to 250,000 students [1]. Moreover, the number of applicants for the Test of Proficiency in Korean (TOPIK) increased from 150,000 in 2012 to 370,000 in 2019 [2]. Many studies and news reports attribute the growth in demand for Korean language learning primarily to *Hallyu (*“Korean Wave”) [[3], [4], [5]], the name given to the growing popularity of Korean pop culture worldwide [6]. Korean pop culture became popular among Asian consumers through the spread of cultural products such as Korean dramas, music, and movies. This spread to North America and Europe and eventually became a global phenomenon [7].

Hallyu has indeed done much to improve South Korea's image, make people interested in learning Korean, and create a significant demand for Korean language classes [5]. However, we cannot ignore the possibility that Hallyu fever may die out [8]. Moreover, although Hallyu is a major reason people want to learn the Korean language, it is unlikely to sustain that interest [4]. For example, Pham and Cao [9] found that Vietnamese students who decided to study Korean in informal language education institutions due to Hallyu immediately abandoned their studies when they lost interest. Therefore, sustaining the international promotion of the Korean language requires the identification of non-Hallyu-related factors that play a decisive role.

Most research on the international promotion of the Korean language takes a linguistic perspective on the composition of Korean language learning motivation, and most studies examine specific countries. The lack of comparative analyses across countries makes it challenging to explain international differences in the results of Korean language promotion, which, in turn, makes it difficult to formulate rational promotion policies. International promotion of the Korean language is an important tool by which South Korea can improve its soft power and achieve foreign policy goals [10] as well as reduce communication problems and misunderstandings in international trade and investment caused by cultural differences [[11], [12], [13]]. We sought to assist in developing such a rational policy using data on TOPIK candidates to investigate the factors that influence the international promotion of the Korean language. This study focuses on the formulation of policies for the international promotion of the Korean language and addresses the following research questions:

RQ1 At the cross-country level, what factors influence the international promotion of the Korean language?

RQ2 What are the reasons for variations in Korean language promotion achievements in different countries?

RQ3 How can we develop effective strategies for the international promotion of the Korean language?

This study makes several important contributions to the existing literature. First, our study is the first to combine linguistic and economic research to provide cross-country-level evidence useful for the sustainable international promotion of the Korean language. Prior studies have provided only individual-level evidence from a linguistic perspective, using questionnaires or interviews targeting specific populations in specific countries. Second, research on language promotion has focused on languages with large numbers of speakers, such as English and Chinese (1.5 billion and 1.2 billion speakers, respectively [14]). Korean, which has 79 million speakers [14], is less commonly taught. This study provides a paradigm that can be applied to research on the international promotion of other, less commonly taught languages.

## 2 Literature review

Currently, research on the international promotion of the Korean language primarily focuses on Korean language learning motivation. Research on Korean language learning motivation can be roughly divided into two categories based on the country in which the research subjects are located. The first category focuses on developed countries, such as Singapore, the United States (US), and Australia. Chan and Chi [15] sought to determine the main factors in Korean language learning motivation for students at the National University of Singapore, using factor analysis to extract five factors from questionnaire data. The five factors were ranked in order of importance as follows: “popular culture, career, achievement, academic exchange, foreign language, and culture.” Lee [5] explored the motivation of students enrolled in beginner Korean language courses at the University of Queensland, Australia, and found that they were motivated primarily by Korean pop culture and secondarily by future job prospects. Lee [3] explored the impact of exposure to Korean pop culture on Korean language learning among learners at the University of Minnesota (US) and found that exposure to Korean pop culture enhanced their motivation to learn Korean. Fraschini and Caruso [16] and Fraschini [17] employed the Q methodology to examine Korean learning motivation among Australian university students and junior and senior high school students by having them imagine themselves as future Korean speakers. Their findings consistently revealed that appreciation of Korean pop culture was a crucial motivator for Korean language learning. Most studies conducted in developed nations have emphasized the value of Korean pop culture in motivating language learners.

The second type of research, conducted in developing countries, produces different results. Gao [18] studied the motivation of non-Korean families in northeast China to attend Korean bilingual schools and found that the economic benefits of business interactions between northeastern China and South Korea increased Korean language learning among non-Koreans. Han [4] studied the motivation for Korean language learning among college students majoring in Korean at three universities in Hanoi, Vietnam, and found that career benefits and income enhancement were more important factors in students’ motivation to learn Korean than other factors such as Korean pop culture.

The aforementioned studies primarily focused on specific populations within specific countries, such as high school and university students, which may only partially represent the overall population of Korean language learners. By contrast, our study utilizes data on TOPIK candidates, which are not limited to any specific group, thereby contributing to a more comprehensive understanding of Korean language learning motivation composition. Moreover, by comparing studies conducted in developed and developing countries, we discovered the distinct characteristics of each country, making it challenging to simply transfer the experiences and policies for promoting the Korean language from one country to another. We might understand the sources of the differences in the achievements of Korean language promotion among countries to develop language promotion policies that align with the specific characteristics of each country. Our study is not confined to individual countries but to multiple countries to find patterns in the international promotion of the Korean language at the cross-country level, thus providing meaningful suggestions for the international promotion of the Korean language.

Regarding the data and methodology, the aforementioned literature relies predominantly on cross-sectional data obtained from questionnaire surveys and interviews. However, cross-sectional data lack the temporal dimension of the variables of interest, resulting in the omission of a substantial amount of information. By contrast, our study utilizes a cross-country panel data model that combines cross-sectional and time-series data. This approach increases the sample size and degrees of freedom and allows for the utilization of fixed effects at different dimensions to mitigate endogeneity issues in regression variables. Furthermore, the data used in this study were sourced from reliable government statistical agencies to ensure credibility.

Compared with the aforementioned literature, we provide new insights at the cross-country level, focusing on the economic perspective. First, as a cross-country study, we believe it is essential to consider the development levels, characteristics, and motivations for learning Korean in different countries to provide targeted recommendations for the international promotion of Korean in each country. Second, this study combines linguistics and economics and finds that economic factors significantly influence people's demand to learn Korean. Additionally, in the conclusion section, we provide more detailed explanations of new insights in the economic, cultural, and educational domains.

## 3 Methodology

### 3.1 Factors affecting the international promotion of Korean (variable selection)

Motivation is a key factor for L2 learning and its maintenance [19]. Motivation is highly correlated with L2 achievement because it motivates positive attitudes during the learning process [20]. L2 motivation can be broadly divided into two categories: integrative and instrumental [21]. In integrative motivation, people learn Korean because of their genuine interest in the language and culture without expecting to gain any benefits. In instrumental motivation, people seek to achieve specific practical goals, such as social and financial benefits, by learning Korean. Using this classification, we analyzed Korean language learners’ motivation at both instrumental and integrative levels to clarify the determinants of the international promotion of the Korean language.

#### 3.1.1 Instrumental motivation for Korean Language learning

Instrumental motivation corresponds to the concept of “foreign-language demand” in economics. It is generally argued that the demand for foreign language learning is motivated by consideration of its costs and benefits [22]. Thus, people decide to learn Korean based on a rational choice meant to maximize their benefits and only if the benefits outweigh the costs.

(1) Benefits of Learning the Korean Language

The benefits of learning Korean derive from the income premium gained from acquiring Korean language skills. According to the human capital theory, the positive effect of foreign language skills on wages comes from the increase in productivity they enable [23]. Productivity increases because foreign language skills allow workers to communicate better if they match the language requirements of the workplace [[24], [25], [26]]. Moreover, according to signaling theory, knowing a foreign language can signal a high sociocultural background to employers, thus indicating higher potential productivity [27]. Therefore, employers who demand labor with specific foreign language skills pay a higher wage premium to workers who have these skills. For example, Liwiński [26] found that foreign language proficiency generated an average wage premium of 11 %.

The demand for labor with foreign language skills is driven by increasing globalization, such as growing international trade and foreign direct investment (FDI) [28]. International trade and FDI depend on verbal and written communication and contracts [29]. Only when both parties can say what they want is a transaction satisfactory for both sides. As tariffs and transport costs decline, language and cultural barriers impose major costs on international transactions such as international trade and FDI [22]. In this context, foreign language skills have become a critical competency demanded by employers. The benefits of foreign language skills are reflected in increased job opportunities and wages. Therefore, we argue that people living in countries with strong trade and investment ties to South Korea have strong incentives to learn Korean. We test this conjecture using data from the international trade and FDI of several countries in South Korea. Referring to Kim and Lee [30], FDI inflows from South Korea as a percentage of gross domestic product (GDP) can be used to measure the investment relationship between South Korea and the sample countries. Based on US Census Bureau criteria, we use the ratio of total two-way trade with South Korea to total trade to measure South Korea's position as a two-way trading partner country in the sample countries. This specifies the trade relationship between the sample country and South Korea.

In addition to the employment demand created by international trade and FDI from South Korea, part of a nation's labor force can migrate to South Korea in search of employment opportunities. The labor market model of immigration suggests that much of the immigration between source and destination countries may depend on the difference in labor income between the two [31]. If the source country's labor income is lower than that of South Korea, the greater the potential gain from immigration to South Korea, the stronger the immigration incentive. The TOPIK certificate is important for immigration to South Korea, as the TOPIK certificate is a requirement for applying for many types of South Korean visas, such as international marriage immigration and work visas [14]. Moreover, proficiency in the official language of the destination country results in a labor income premium of 5–35 % for international immigrants [32]. Therefore, we argue that the stronger the motivation of a country's residents to migrate to South Korea, the greater the number of residents who wish to learn Korean. According to Abbott and Silles [33], the ratio of the real per capita GDP of the destination country to the real per capita GDP of the immigrant's home country can explain wage differentials between the two economies, thus representing the economic incentives for immigration. Therefore, we used the ratio of a country's real per capita GDP to South Korea's real per capita GDP as a measure of the benefits that residents of that country can gain by migrating to South Korea to verify the impact of the motivation to migrate to South Korea on the international promotion of Korean.

Furthermore, the motivation for studying in South Korea is instrumental because international students come to South Korea to increase their human capital and improve labor market outcomes [33]. To enter most South Korean university degree programs, international students usually need to obtain a TOPIK level of three or higher and graduate with a TOPIK level of four or higher [34]. Therefore, we argue that the greater the demand among residents of a country to study in South Korea, the greater the number of residents who wish to learn Korean. Beine et al. [35] used the number of international students studying to measure the attractiveness of each country to international students. Similarly, we measured the demand to study in South Korea in each country as the number of international students from that country studying at South Korean schools and examined how the motivation to study in South Korea impacts the promotion of the Korean language.

(2) Cost of Learning the Korean Language

Having summarized the potential benefits of learning Korean, we should also consider its costs. This cost originates from several sources.

The availability of appropriate educational resources is a prerequisite for learning Korean. The King Sejong Institute, established in 2007 and supported by the Korean government, provides Korean language education resources. As of June 2021, the King Sejong Institute had 234 institutions in 82 countries [36]. It offers many free Korean language and cultural courses [36]. Therefore, we posit that establishing the King Sejong Institute contributed to promoting the Korean language. The Internet, which is now widely used as an educational tool [37], offers many online Korean language courses [30]. Hence, residents of countries with more developed Internet infrastructure have more access to the resources required to learn Korean [30]. Following the Choi and Hoon Yi [38] approach to constructing variables, we used the percentage of Internet users in the total population as a variable to measure the impact of the Internet on Korean promotion.

Second, the difficulty of learning Korean is an important cost. This difficulty varies depending on the native language of the learner because the nature of the differences between a given language and Korean is language-specific. The greater the difference between the learners' native language and Korean, the greater the time and effort required to learn the language [39]. Therefore, the more difficult it is for a country's residents to learn the Korean language, the fewer the residents who are likely to want to learn it [39]. However, the fewer the number of Korean speakers in a country, the greater the benefits that Korean speakers can gain through oligopolistic pricing power, which will, in turn, increase the need for residents of that country to learn Korean [29,40]. Therefore, we cannot predict how the difficulty of learning Korean will impact the demand for Korean language learning. It is also difficult to quantitatively measure the difficulty of learning Korean among learners of various native languages. However, to prevent this omitted variable from influencing our empirical results, we controlled for unobservable features across different language groups by including language-group fixed effects in our model.

Finally, geographic distance is also an important cost of learning the Korean language because the greater the distance, the weaker the desire for cultural exchange and cooperation between countries [12]. At the same time, the cost of international labor mobility is related to the physical distance between countries, and the cost of migration increases as distance increases [31]. Therefore, the more distant a country is from South Korea, the lower the demand in that country for migration to South Korea, and thus, the lower the demand for Korean language learning. We used the straight-line distance between capital cities as a representative variable to measure the physical distance between countries. This method of constructing variables has been widely employed in international migration [31,33], international cultural cooperation [22], and language economics [41].

#### 3.1.2 Integrative motivation for Korean Language learning

**Fig. 1:**
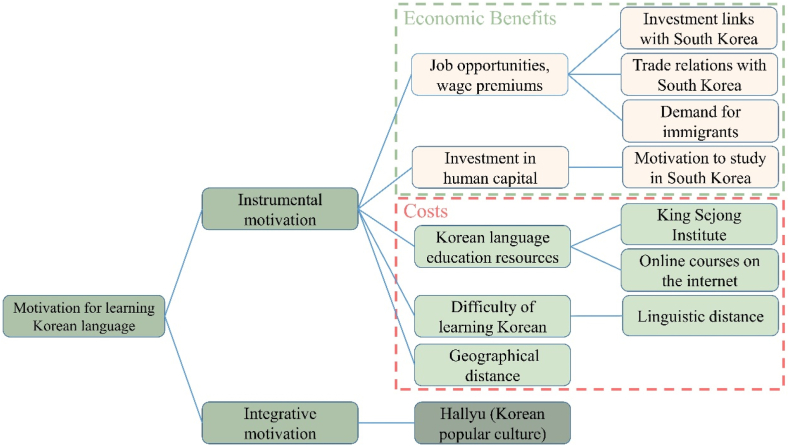
Factors determining the international promotion of the Korean language.

Learners driven by integrative motivation learn the target language out of a genuine interest in the target language, related cultures, and people [42]. Hallyu has been the most critical factor in the integrative motivation to learn Korean. For example, Chan and Chi [15], Lee [5], and Lee [3] studied the motivation to learn Korean at the University of Singapore, the University of Minnesota, and the University of Queensland, respectively, finding that Korean pop culture was the primary factor motivating students to learn Korean. However, these results were derived from questionnaires and interviews. No study has used specific Hallyu-based data to consider the impact of Hallyu on the international promotion of Korean. Therefore, we compiled data on the export of South Korean cultural goods as a proxy for Hallyu, based on UNESCO's definition of “cultural commodities” (as specified in the HS codes). Hallyu variables have been widely used in Hallyu [[43], [44], [45]]. The factors involved in this process are illustrated in Fig. 1.

### 3.2 Data

The TOPIK is a Korean language test administered by the National Institute for International Education (NIIED) of the Korean Ministry of Education. It assists foreigners and expatriates whose native language is not Korean in learning the language and helps expand its popularity [2]. The TOPIK test score can be used as a standard for measuring and evaluating the ability to use Korean and is widely used in education in South Korea, visa applications (such as for permanent residency or work in South Korea), and employment in South Korean companies or public institutions [2]. The TOPIK has been administered for 25 years and is the leading and most influential international Korean language proficiency test, with 242 test sites across 86 countries [2]. Most foreign language learners take language proficiency tests as part of their learning [12]. Hence, the number of candidates who undergo a language proficiency test is a measure of the popularity of the language being tested [12]. Xie [12] used data on Chinese proficiency test candidates to examine the relationship between the international promotion of Mandarin and increasing income in China. Similarly, we used TOPIK candidate data from various countries to measure the popularity of Korean. Specifically, we used the number of TOPIK test applicants as an indicator of the results of the international promotion of the Korean language as well as the number of test-takers and successful test-takers in a robustness test.

**Fig. 2:**
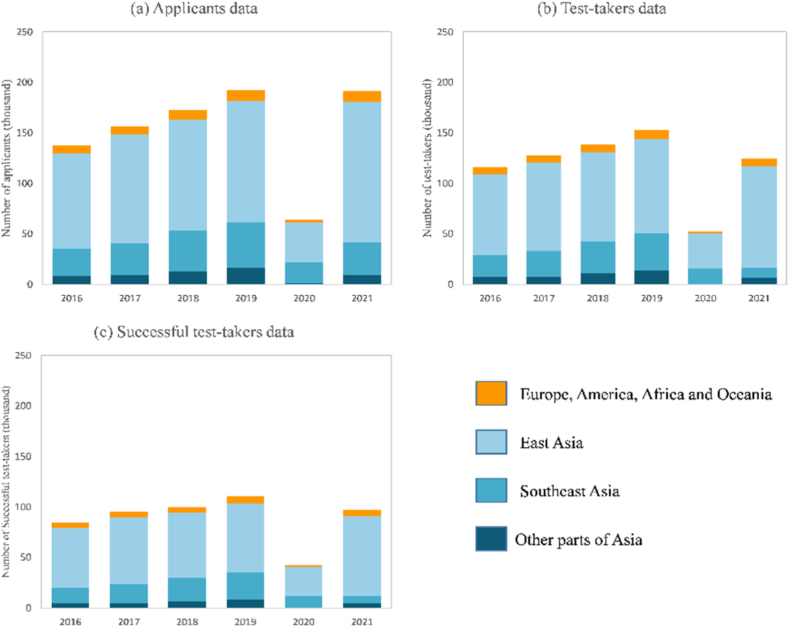
TOPIK test candidate data.

As Fig. 2 shows, the TOPIK candidates from 2016 to 2022 were concentrated in East and Southeast Asia, whereas other areas exhibited a reasonably significant growth trend, although the number of candidates was minimal. Therefore, the international promotion of the Korean language requires more effort, especially outside Asia.

We analyzed data on global (non-South Korean) TOPIK candidates from 2016 to 2019. Data from 2020 to 2021 may not be representative because many tests were postponed owing to government control measures during the COVID-19 pandemic and had not been completely restored by 2021. In addition, we excluded countries that began the test between 2016 and 2019 and did not offer the test consecutively because these countries may have accumulated more than two years’ worth of Korean language learners who wished to take the TOPIK test in any given year; using TOPIK candidate data would have overestimated the degree of Korean language promotion measured in these countries for a year.

As indicated in Section 3.1, we summarized the factors that may affect the international promotion of Koreans and generated the related variables to test their effects. Table 1 and Table 2 present the variables’ definitions and descriptive statistics.

**Table 1:** Dependent variable descriptions and descriptive statistics.

Variables	Definition	Meaning	Mean	Std. Dev.
Y	Number of TOPIK test applicants	Results of international promotion of the Korean language	2349	9444
Y_R1	Number of TOPIK test-takers	Robustness test	1901	7620
Y_R2	Number of successful TOPIK test-takers	Robustness test	1373	5439
Y_D	Dummy variable, 1 for holding TOPIK test, 0 otherwise.	For the selection equation in the Heckman two-step method	0.388	0.487

**Table 2:** Independent variable descriptions and descriptive statistics.

X variables	Definition	Meaning	Mean	Std. Dev.
FDI	Ratio of country's FDI from South Korea (USD) to country's GDP (USD million)	Investment relations with South Korea	0.762	3.557
Trade	Country's trade with South Korea as a percentage of the country's total trade	Trade relations with South Korea	4.078	13.87
Income	South Korea's real GDP per capita/country's real GDP per capita	Benefits of immigrating to South Korea	11.88	17.03
Distance	Distance from country's capital to capital of South Korea (km)	Cost of immigrating to South Korea	9599	3733
Education	Number of international students from country in South Korea	Demand for education in South Korea	757.9	5501
Internet	Percentage of Internet users in population	Degree of development of Internet infrastructure	55.54	27.74
KSI	Number of King Sejong institutes per million people	Korean language education resources provided by King Sejong Institute	44.94	135.5
Hallyu	Per capita consumption of Korean cultural goods (USD)	Popularity of Hallyu	0.084	0.326
Population	Total population (millions)	Control for impact of population	41.44	153.5
Z variable				
FTA	Dummy variable; 1 for FTA with South Korea, 0 otherwise	Meet valid exclusion restriction	0.296	0.457

The TOPIK candidate data were obtained from the National Institute for International Education (NIIED). Data on South Korea's international trade and cultural exports were obtained from the South Korean Customs Service, whereas data on the King Sejong Institute were obtained from the King Sejong Institute Foundation. We also used distance data from Google Maps; international student data from the South Korean Ministry of Education; FTA (Free Trade Agreement) data from the South Korean Ministry of Industry, Trade, and Energy; and other data from the World Bank.

### 3.3 Model

Cross-country panel data models have been extensively used to study the international promotion of languages. For example, Xie [12] used a cross-country panel data model to investigate the relationship between increasing China's income and the international promotion of the Chinese language. Kim and Lee [30] used a cross-country panel data model to investigate factors affecting English language proficiency among residents of over 60 countries at the cross-country level. The popularity of cross-country panel data models in applications primarily lies in the several advantages they offer because of their characteristics, including the provision of richer information, increased variability, reduced collinearity among variables, greater degrees of freedom, and enhanced efficiency [46]. A more salient advantage is that the analysis of panel data allows us to consider not just one country but many others, and the use of variation between countries over time provides a more robust estimate of the empirical results [47]. In summary, this study constructs a cross-country panel data model to investigate the factors that determine the international promotion of the Korean language as well as the effect of sample selectivity bias and the underlying cross-country panel data model, thus choosing the Heckman two-step approach to estimate our model.

**Fig. 3:**
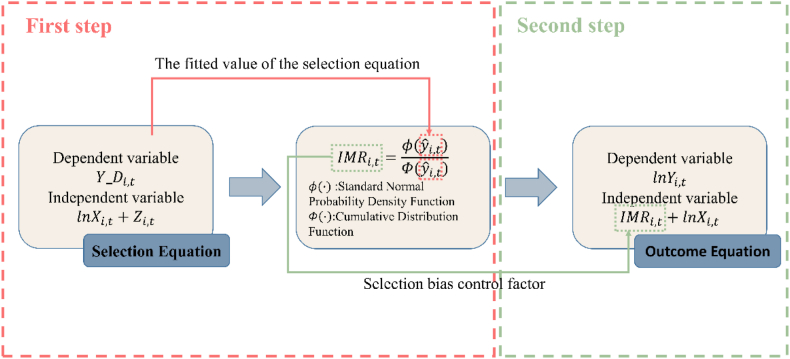
Heckman two-step method.

As not all countries held TOPIK exams, our data included many missing values (i.e., for countries that did not hold TOPIK exams). The TOPIK test data contain two types of information relevant for analyzing the factors affecting the international promotion of the Korean language:1) whether the TOPIK test was held, and 2) the number of TOPIK test applicants. Using ordinary least squares (OLS) regression analysis only for the latter information type would exclude countries that did not conduct TOPIK tests, which would lead to a sample selection bias and affect the consistency of the estimated coefficients. Therefore, the two-step Heckman method was selected [48]. The first step involved building a probit model (selection equation) to measure the decision to conduct the TOPIK test. The selection equation was then used to construct the inverse Mills ratio (IMR), which was added to the outcome equation in the second step to control selection bias. The Heckman two-step method is illustrated in Fig. 3.

The selection equation for the first stage, estimated using a binary probit regression model, is given in Equation (1):

(1) $$\begin{array}{c} {Y_{D}}_{i,t}=\alpha +{\beta lnX}_{i,t}+\gamma Z_{i,t}+\varepsilon_{i,t} \end{array}$$

where ${Y\_D}_{i,t}$ is a dichotomous variable set to one if the country holds the TOPIK test and zero otherwise. $X_{i,t}$ represents the factors that influence a country's decision to conduct a TOPIK test. To address possible heteroscedasticity, we use logarithms for all variables except the dummy variables. β and γ are the coefficients of the variables, and α is a constant term. $\varepsilon_{i,t}$ is a random error term.

Valid identification of the Heckman model relies on one of two conditions: the presence of a valid exclusion restriction in the selection equation or the presence of data that satisfy the assumption of a normal distribution. Wolfolds and Siegel [49] demonstrated that the Heckman method is usually less reliable than OLS if the regression model does not satisfy the exclusion restrictions. Meeting the valid exclusion restriction was crucial for our model because of the fragility of relying on the assumption of normality. The valid exclusion restriction requires us to include variable $Z_{i,t}$ in the selection equation, which only affects whether the TOPIK test is conducted (and not the number of applicants). The variable $Z_{i,t}$ is used only in the selection equation, and not as an independent variable in the outcome equation. The $Z_{i,t}$ variable is a dummy variable that reflects an FTA with South Korea. Assuming that an FTA represents closer economic and political ties, we posit that countries that sign an FTA with South Korea are more likely to undergo TOPIK tests. However, the FTA had a negligible effect on the number of TOPIK test applicants. Although an FTA can affect the number of TOPIK test applicants by increasing international trade and FDI, we control for this effect using appropriate variables.

The outcome equation for the second step is as follows:

(2) $$\begin{array}{c} {lnY}_{i,t}=\alpha +{\beta lnX}_{i,t}+{\theta IMR}_{i,t}+u_{i}+d_{t}+\varepsilon_{i,t} \end{array}$$

where the dependent variable $Y_{i,t}$ in the outcome equation is the number of TOPIK test applicants, and the independent variable $X_{i,t}$, as in the selection equation, represents factors that may affect the number of TOPIK test applicants. Including ${IMR}_{i,t}$ in the outcome equation solves the sample selection bias problem. α and $\varepsilon_{i,t}$ represent the intercept and error terms, respectively.

Because languages within the same language group are similar, learning a foreign language in the same group as the native language is easier than learning a foreign language in a different language group. Korean does not belong to the same language group as the official languages of the sample countries. Therefore, language group fixed effects can effectively control the impact of language distance and language learning difficulty. Thus, we first divided the official languages of the countries in the sample into 21 groups. When a country had multiple official languages, the language with the largest population share was selected as the representative language. The dummy variable $u_{i}$, corresponding to each language group, was then added to the model (there were 20 dummy variables due to multicollinearity) to control for unobservable characteristics across language groups, including language distance (i.e., language learning difficulty). Given the dimensionality of the panel data, we also included time-fixed effects $d_{t}$ in the model to control for global factors affecting all countries simultaneously.

## 4 Results

### 4.1 Benchmark regressions

Table 3 reports the estimation results of the Heckman two-step and OLS methods for the determinants of the international promotion of the Korean language. In the selection equation results, the coefficient of FTA is statistically significant, proving that it is a valid instrument that meets the exclusion restriction condition. In the outcome equation results, the coefficient of IMR is statistically significant, indicating a significant sample selection bias in the OLS regression, which needed to be addressed using the Heckman two-step method. After controlling for sample selection bias with the IMR variable, the regression coefficients for the other variables in the outcome equation provided the desired results.

**Table 3:** Benchmark regression results.

Variables	Heckman Two‐step Selection Equation	Heckman Two‐step Outcome Equation	OLS
FDI	$0.378^{**}$ (0.190)	$0.756^{***}$ (0.151)	$0.509^{***}$ (0.1440
Trade	0.006 (0.077)	$0.119^{**}$ (0.058)	$0.172^{**}$ (0.057)
Income	$0.298^{**}$ (0.122)	$0.503^{***}$ (0.087)	$0.389^{***}$ (0.081)
Distance	${-0.831}^{***}$ (0.248)	${-1.147}^{***}$ (0.260)	${-0.842}^{***}$ (0.263)
Education	$0.352^{***}$ (0.084)	$0.492^{***}$ (0.068)	$0.348^{***}$ (0.081)
Internet	$1.274^{***}$ (0.244)	$1.227^{***}$ (0.254)	$0.673^{***}$ (0.172)
KSI	$0.118^{***}$ (0.035)	$0.217^{***}$ (0.026)	$0.159^{***}$ (0.236)
Hallyu	−0.002 (0.521)	$0.978^{***}$ (0.371)	$1.040^{***}$ (0.430)
Population	$0.491^{***}$ (0.095)	$0.386^{***}$ (0.067)	$0.222^{***}$ (0.055)
FTA	$0.386^{*}$ (0.208)	−	−
IMR	−	$1.194^{***}$ (0.287)	−
Constant	${-7.630}^{***}$ (2.168)	−0.645 (2.271)	3.118 (2.350)
LanguagegroupFE	Y		Y
YearFE	Y		Y
Obs.	676		264
$R^{2}$	–		0.871

Robust standard errors are indicated in parentheses. *p < 0.1; **p < 0.05; ***p < 0.01.

Among the variables describing the benefits of instrumental motivation, the estimated coefficients on FDI and Trade are positive and statistically significant. This result suggests that a country with close trade and economic ties to South Korea contributes to the promotion of the Korean language. Specifically, firms engaged in international business activities involving South Korea need employees with Korean language skills who are willing to pay a wage premium for them and who are willing to learn Korean when motivated by economic benefits. Vietnam hosts 3234 Korean companies. In Vietnam, there is a saying that goes, “Knowing English doubles your salary; knowing Korean triples it [50]." With the influx of Korean enterprises into Vietnam, the ability to speak Korean has been regarded as a guarantee of employment. Moreover, companies are willing to offer higher wages to individuals proficient in Korean, which has led to increased interest in learning the language [50]. Xie [12] found similar results in his study on the international promotion of the Chinese language, revealing that FDI and trade from China were important factors.

Generally, if the country's average income is lower than that of South Korea, all other things being equal, the country's residents will have a stronger motivation to migrate to South Korea [33]. Because Korean language skills are beneficial and often required for immigration to South Korea, the stronger the motivation to migrate to South Korea, the stronger the motivation to learn Korean. This hypothesis is confirmed by the coefficient of Income, which is significantly positive, indicating that the demand for immigration and demand for Korean language learning are positively related.

Similarly, the effect of motivation to study in South Korea on the promotion of Korean language proficiency was confirmed. Education has a positive and statistically significant coefficient, indicating that the increased demand for studies in South Korea leads to an increased demand for Korean language learning. South Korean higher education is increasingly globally competitive, and with the support of the government, South Korea is expected to become the center of education in Asia [51]. Consequently, South Korea has attracted many international students through its higher education resources. As the Korean language is often required to live and study in South Korea, the demand for South Korean education is naturally accompanied by the demand for Korean language learning.

Among the variables describing the cost of instrumental motivation, the coefficients of KSI and Internet are significantly positive, indicating that Korean language learning resources are an important factor in determining Korean international promotion. The lack of Korean language learning resources has prevented some people who want to learn the language from doing so. Therefore, providing more Korean learning resources is an important way to promote Korea internationally. Using Vietnam as an illustrative case, the burgeoning economic ties between Vietnam and South Korea have engendered substantial demand for individuals proficient in the Korean language. This demand stems from the realization that competency in the Korean language can yield enhanced employment prospects and higher remuneration rates for Vietnamese individuals. A noteworthy development in 2021 was the Vietnamese government's official designation of Korean as its primary foreign language. Furthermore, for two consecutive years, the Department of Korean Studies at the University of Social Sciences and Humanities in Hanoi set the admission cutoff at the maximum achievable score [50]. These remarkable circumstances underscore the intense competition within Vietnam for superior Korean language education resources. Consequently, augmenting the availability of educational provisions would significantly bolster the global promotion of the Korean language. In addition, the effect of language distance (language learning difficulty) was examined using language-group fixed effects. Similar to Xie [12], we find that geographic distance is an important factor hindering international language promotion.

The Hallyu coefficient, a proxy for integrative motivation, was significantly positive, suggesting that Hallyu is a key motivating factor in Korean-language learning, as highlighted in previous studies. On January 17, 2023, CNN published an article titled, “South Korea brought K-pop and K-dramas to the world. Korean could be the next choice.” The report highlights Korean as one of the fastest-growing languages globally, surpassing traditionally popular languages such as Chinese in foreign language markets across multiple countries. This reflects the influence of the Hallyu [52]. In addition to presenting individual-level evidence offered by prior research, we are the first to use economic data to identify Hallyu's contribution to the international promotion of Korean at a cross-country level.

### 4.2 Robustness tests

#### 4.2.1 Substitution of dependent variables

To verify the robustness of our results, we replaced the dependent variables with the number of total TOPIK and successful TOPIK test-takers. The results in Table 4 show that the coefficient magnitudes and statistical significance levels of the variables do not change significantly, confirming the robustness of our main estimation results.

**Table 4:** Robustness test results.

Variables	Outcome Equation (Number of total TOPIK test-takers)	Outcome Equation (Number of successful TOPIK test-takers)
FDI	$0.746^{***}$ (0.150)	$0.784^{***}$ (0.152)
Trade	$0.123^{**}$ (0.057)	$0.108^{*}$ (0.060)
Income	$0.505^{***}$ (0.087)	$0.483^{***}$ (0.087)
Distance	${-1.140}^{***}$ (0.259)	${-0.977}^{***}$ (0.257)
Education	$0.493^{***}$ (0.065)	$0.511^{***}$ (0.062)
Internet	$1.247^{***}$ (0.254)	$1.315^{***}$ (0.263)
KSI	$0.209^{***}$ (0.026)	$0.204^{***}$ (0.026)
Hallyu	$1.044^{***}$ (0.386)	$0.999^{**}$ (0.417)
Population	$0.369^{***}$ (0.065)	$0.347^{***}$ (0.062)
IMR	$1.185^{***}$ (0.278)	$1.240^{***}$ (0.266)
Constant	−0.643 (2.285)	−2.230 (2.369)
LanguagegroupFE	Y	Y
YearFE	Y	Y
Obs.	676	676

Robust standard errors are indicated in parentheses. *p < 0.1; **p < 0.05; ***p < 0.01.

#### 4.2.2 Multicollinearity test

The high correlation between explanatory variables is known as multicollinearity, which leads to an increase in variance and thus affects confidence intervals and hypothesis testing [53]. We use the variance inflation factor (VIF) to examine the presence of multicollinearity in the model. Table 5 presents the results, demonstrating that all explanatory variables exhibited VIF values below the threshold of 5, indicating the absence of multicollinearity in the analyzed model.

**Table 5:** Multicollinearity test result.

Variable	VIF
FDI	1.39
Trade	1.53
Income	2.93
Distance	2.43
Education	4.27
Internet	2.59
KSI	1.29
Hallyu	1.46
Population	2.20
MeanVIF	2.23

#### 4.2.3 Instrumental variable regression analysis

Hallyu, the King Sejong Institute, and the international promotion of the Korean language are all associated with the characteristics of the Korean language and culture. Therefore, the correlations between them may be attributed to unobserved factors at the Korean language and cultural levels. Thus, if omitted variables are correlated with one or more explanatory variables in the model and correlated with the dependent variable, endogeneity is introduced, leading to biased estimates and potentially spurious correlations between the explanatory and dependent variables [54]. Additionally, reverse causality in the model is a source of endogeneity. For example, one of the purposes of establishing King Sejong Institutes is to meet the local demand for Korean language learning. Therefore, a higher demand for Korean language learning will lead to the establishment of more King Sejong Institutes. Thus, there may be bidirectional causality between the King Sejong Institute and the international promotion of the Korean language. We thus used an instrumental variable approach to control for endogeneity issues and test whether the presence of endogeneity bias affects the robustness of our results. Specifically, we run a two-stage least-squares regression after selecting instrumental variables consistent with correlation and exogeneity (for a more detailed description of the instrumental variables approach, see Wooldridge [55]). We explain the instrumental variables corresponding to King Sejong Institute and Hallyu below.

Regarding the King Sejong Institute, Eom et al. [56] examined the Korean government's strategic placement of King Sejong Institutes and found that the Korean government uses King Sejong Institutes as a complement to public diplomacy, in addition to considering local needs for Korean language education, when selecting countries to establish King Sejong Institutes. Considering that high levels of official development assistance (ODA) are a concrete manifestation of South Korea's diplomatic offensive, it is more likely that the King Sejong Institute will be established in a country that receives high levels of ODA from South Korea [56]. We chose the amount of ODA received from South Korea per capita in our sample countries as the instrumental variable. Using ODA as an instrumental variable permits us to isolate the influence of diplomatic and political factors that determined the establishment of the King Sejong Institute, which is distinct from the impact of Korean language learning demands and cultural factors.

We used the ratio of imported cultural goods to GDP as a measure of the sample countries' preferences for consuming foreign cultural products and employed it as an instrumental variable. Logically, the higher the consumer preference for foreign cultural products in the sample countries, the more likely they are to consume greater quantities of cultural goods from South Korea (Hallyu). Moreover, no direct causal relationship exists between consumption preferences for foreign cultural products and the international promotion of the Korean language. This is substantiated by the fact that cultural products from South Korea constituted only 2.48% of global exports of cultural goods in 2019, as reported by UNESCO, despite their remarkable growth.

Table 6 presents the regression results obtained by using the instrumental variables approach. The results of the first-stage regression reveal that the instrumental variable, South Korea's ODA, exhibits a statistically significant positive effect at the 1 % level. This suggests that it promoted the establishment of the King Sejong Institute, which is consistent with the empirical findings of Eom et al. [56]. The significant positive coefficient of the instrumental variable, representing the preference for foreign cultural products, confirms our reasoning that countries with a higher preference for foreign cultural products are more likely to consume a greater quantity of cultural products from South Korea. The first-stage F-values for both regressions are greater than 10, indicating the absence of a weak instrumental variable problem; that is, the instrumental variables are highly correlated with the endogenous variables [57]. Thus, the selected instrumental variables were appropriate. In the regression results of the second stage, the estimated coefficient for King Sejong Institute is 0.311, which is slightly higher than the coefficient of 0.217 obtained in Table 3 of the Heckman two-step outcome equation. This suggests that the presence of endogeneity underestimates the role of the King Sejong Institute. The coefficient for Hallyu was 3.422, which is significantly higher than the coefficient of 0.978 in the Heckman two-step outcome equation. This indicates that, without correcting for endogeneity, the impact of Hallyu on the international promotion of Korean would be severely underestimated. However, even without correcting for endogeneity, the results demonstrate the promotional effects of the Hallyu and King Sejong Institute, once again confirming the robustness of the empirical findings.

**Table 6:** Instrumental variable regression results.

Variables	(1)	Variables	(2)
Second-stage regression results			
KSI(Endogenousvariable)	$0.311^{**}$ (0.123)	Hallyu(Endogenousvariable)	$3.422^{*}$ (1.789)
Controlvariables	Y	Controlvariables	Y
LanguagegroupFE	Y	LanguagegroupFE	Y
YearFE	Y	YearFE	Y
Obs.	264	Obs.	248
$R^{2}$	0.851	$R^{2}$	0.881
First-stage regression results			
ODA(Instrumentalvariable)	$0.217^{***}$ (0.068)	CG(Instrumentalvariable)	$4.022^{***}$ (0.913)
$F_{1}$	10.243	$F_{1}$	19.404

Note: 1. $F_{1}$ represents the F-statistic of the first stage.

2. ODA data from ODA Bureau, Office for Government Policy Coordination, Korea.

3. Data on the value of imported cultural goods (CG) as a percentage of GDP are from UNESCO.

#### 4.2.4 Sensitivity analysis

If unobserved confounders are correlated with both the explanatory and dependent variables, our estimated relationship may suffer from omitted variable bias. Therefore, we employed the sensitivity analysis framework developed by Cinelli and Hazlett [58] to assess the impact of unobserved confounders on our results. We assume the presence of a confounding factor that has three times the effect of Hallyu and then examine the sensitivity of the variable coefficients in the Heckman two-step outcome equation of the baseline regression results presented in Table 3. We used the Hallyu variable as the benchmark because its role in promoting the internationalization of the Korean language has been widely acknowledged in the news media and research in Korean language education. Table 7 presents the sensitivity analysis results. The findings indicate that, even in the presence of confounding factors with an intensity three times that of the Hallyu variable, the coefficients of the other explanatory variables remain statistically significant at the 5 % level (i.e., their 95 % confidence intervals do not include zero), except for the trade variable. Given the difficulty in enumerating omitted variables with an intensity three times that of the Hallyu variable, it is unlikely that the results of our empirical analysis, except for the trade variable, are significantly affected by omitted variable bias.

**Table 7:** Sensitivity analysis results.

Unobserved confounders	Treatment variable	Coefficient	Standard error	t-statistic	Lower CI (95 %)	Upper CI (95 %)
3×Hallyu	FDI	0.425	0.177	2.408	0.077	0.773
3×Hallyu	Trade	0.051	0.064	0.801	−0.075	0.178
3×Hallyu	Income	0.453	0.081	5.604	0.294	0.612
3×Hallyu	Distance	−0.932	0.238	−3.924	−1.400	−0.464
3×Hallyu	Education	0.475	0.061	7.855	0.356	0.594
3×Hallyu	Internet	1.190	0.197	6.042	0.802	1.578
3×Hallyu	KSI	0.191	0.026	7.297	0.140	0.243
3×Hallyu	Population	0.348	0.057	6.143	0.236	0.460

Note: IC = confidence interval.

The results for trade variables are not robust, which may be attributed to the characteristics of international trade. Although learning Korean can reduce the costs associated with trading with South Korea and lead to economic benefits, international trade is often characterized by its short-term and transactional nature. Consequently, in situations where long-term relationships between buyers and sellers are lacking, buyers tend to opt for a third language, such as English, to facilitate their transactions [59]. Thus, the promotion of Korean language learning is influenced primarily by international trade, which involves long-term relationships. However, we cannot differentiate between long- and short-term relationships in international trade using the available data, which undermines the robustness of the trade variable results.

### 4.3 Heterogeneity analysis

The results of previous studies on Korean language learning motivation suggest that the factors influencing the international promotion of Koreans may depend on the level of development of the target country. In developed countries, Korean-language learners are more motivated by Hallyu than employment or income. By contrast, Korean language learners in developing countries are more concerned about employment and income. Therefore, to test whether the international promotion of the Korean language is affected by differences in development levels between the sample countries, we divided the sample into two subsamples, representing developed and developing countries, according to the IMF definition of “developed economy.” We then conduct regression analyses on each of the two subsamples. Table 8 presents the results of the study. The following descriptions of the variable coefficients are based on the results of the outcome equations for comparative purposes:

**Table 8:** Heterogeneity analysis results.

Variables	Outcome Equation (Developed countries)	OLS (Developed countries)	Outcome Equation (Developing Countries)	OLS (Developing Countries)
FDI	$0.737^{***}$ (0.173)	$0.653^{***}$ (0.167)	0.172 (0.180)	0.053 (0.178)
Trade	$0.482^{*}$ (0.243)	0.390 (0.241)	0.114 (0.076)	$0.186^{**}$ (0.072)
Income	1.470 (0.891)	1.168 (0.886)	$0.333^{***}$ (0.090)	$0.421^{***}$ (0.087)
Distance	${-2.037}^{***}$ (0.545)	${-1.939}^{***}$ (0.565)	${-1.225}^{***}$ (0.246)	${-1.254}^{***}$ (0.276)
Education	$0.660^{***}$ (0.165)	$0.633^{***}$ (0.162)	$0.329^{***}$ (0.079)	$0.224^{**}$ (0.093)
Internet	0.296 (1.252)	0.0780 (1.256)	$0.806^{***}$ (0.191)	$0.723^{***}$ (0.187)
KSI	$0.221^{**}$ (0.087)	$0.218^{**}$ (0.091)	$0.203^{***}$ (0.027)	$0.220^{***}$ (0.028)
Hallyu	$1.001^{*}$ (0.573)	$1.201^{**}$ (0.595)	−0.141 (0.420)	−0.240 (0.408)
Population	$0.495^{***}$ (0.095)	$0.472^{***}$ (0.135)	$0.298^{***}$ (0.054)	$0.263^{***}$ (0.058)
IMR	0.714 (0.467)	−	$0.603^{***}$ (0.215)	−
Constant	$9.180^{*}$ (4.916)	$9.753^{*}$ (5.268)	$3.566^{*}$ (2.135)	5.429 (2.368)
LanguagegroupFE	Y	Y	Y	Y
YearFE	Y	Y	Y	Y
Obs.	124	60	552	204
$R^{2}$	−	0.966	−	0.887

Robust standard errors are indicated in parentheses. *p < 0.1; **p < 0.05; ***p < 0.01.

Consistent with expectations, Hallyu's influence was statistically significant only in developed countries, indicating that Hallyu's contribution to the international promotion of Korean is more important in developed countries than in developing countries. For example, in recent years, influenced by Hallyu, there has been a global trend to study the Korean language and culture, and the US is no exception. According to the Modern Language Association of America, the number of students studying Korean at universities in the US was approximately 10,000 in 2002 but increased to 17,100 in 2016, indicating a remarkable growth of 68 % [60].

In terms of employment and income, the effect of income disparity is consistent with expectations because the coefficient of Income is significantly positive only in the sample of developing countries. Per capita income in developed countries is higher and closer to that in South Korea; therefore, residents’ Korean language learning is less influenced by the economic benefits of migrating to South Korea. In addition, the lower benefits of migrating to South Korea could make the effects of distance and the cost of moving seem greater for these residents. Thus, distance has a much more significant impact on developed countries than on developing ones.

Surprisingly, the FDI and Trade variables, which also represent employment and income, are significantly positive in the developed-country sample but not in the developing-country sample. This result may be related to differences in the characteristics of international business activities conducted by South Korea in countries with different levels of development. For example, Lee [61] finds that South Korean FDI flowing into developing countries is cost-oriented, searching for cheaper labor costs, whereas South Korean FDI flowing into developed countries is market-oriented and aims at market expansion [61]. Moreover, as consumers of South Korean products are residents, localization is vital to market-oriented FDI [62]. Therefore, South Korean companies conducting business in developed countries are more interested in hiring local talent to achieve localization success than South Korean companies in developing countries. In other words, South Korean companies conducting business in developed countries are willing to provide more employment opportunities and higher wage premiums to local workers with Korean language skills.

The coefficient of Internet is significantly positive in developing countries and not significant in developed countries. This finding implies that Korean language education resources are important in developing countries and that Korean language education resources are lacking in developing countries. This result also explains why the coefficient of Education is larger in the developed-country sample than in the developing-country sample. Owing to the abundance of Korean language education resources, students in developed countries are given more opportunities to learn Korean before arriving in South Korea than students in developing countries. In addition, we found similar impacts of the King Sejong Institute in developed and developing countries. Therefore, the role of the King Sejong Institute in expanding the international promotion of the Korean language does not exhibit heterogeneity based on the level of national development.

## 5 Conclusion

This study is the first to combine linguistics and economics to analyze the factors determining the international promotion of the Korean language at the cross-country level. Using the classification of L2 motivation in linguistics and the concept of “costs and benefits” in economics, we compiled the factors that may affect the international promotion of Korean. We estimate the importance of these factors using TOPIK candidates and economic data from 2016 to 2019. Our estimation analysis uses the Heckman two-step method, which effectively addresses the problem of sample selection bias.

Our main findings are as follows: In terms of instrumental motivation, we find that close trade and economic ties with South Korea, motivation to migrate to South Korea, and motivation to study in South Korea are important drivers of the international promotion of Korean, whereas the lack of Korean language learning resources is a significant hindrance. In terms of integrative motivation, we confirmed, for the first time, Hallyu's contribution to the international promotion of the Korean language using econometrics.

We ensure the robustness of our main findings through the following efforts. First, we replace the dependent variables with the number of total TOPIK and successful TOPIK test-takers and find that the results remain unchanged. Second, considering that the King Sejong Institute, Hallyu, and the international promotion of the Korean language might all be influenced by factors related to the Korean language and culture, this brings about endogeneity problems, namely spurious correlation and reverse causality. We address this endogeneity by using instrumental variable regression analysis, and the results once again affirm the promotional role of Hallyu and the King Sejong Institute in the international promotion of the Korean language. Third, we employ the variance inflation factor (VIF) to demonstrate that multicollinearity does not affect our results. Fourth, due to data availability, we do not consider other potential determinants that might influence the international promotion of the Korean language, which could lead to an omitted variable bias. We assess the impact of this bias using sensitivity analysis and find that, other than the trade variable, our empirical results are unlikely to be significantly influenced by omitted variable bias.

We also find that a nation’s development level affects the importance of the factors influencing Korea’s international promotion. For developed countries, Hallyu is the most significant factor, followed by international business and motivation to study in South Korea. Learning resources and motivation to migrate to South Korea is crucial for developing countries.

We believe that the results of this study provide the following insights into the formulation of South Korea's international promotion policies for the Korean language: First, in terms of economic policy, Korea's international promotion relies on the international business activities of Korean companies. Commercial activities between a country and Korean companies can generate demand for Korean language learning among its citizens. The Korean government can provide Korean language education resources to local employees of Korean companies, thereby assisting in the successful implementation of localization strategies while promoting the Korean language. Second, in terms of cultural policy, Hallyu can stimulate foreign interest in learning Korean. South Korea can combine Korean language instruction with Hallyu content by establishing more Sejong Institutes and Korean Cultural Centers in various countries, thus enhancing the efficiency of the international promotion of the Korean language. Third, regarding educational policy, South Korea's international promotion can be combined with policies aimed at attracting international students. We recommend providing Korean language education resources to students who wish to study in South Korea prior to their arrival, especially to students from developing countries where access to online resources may be limited. For example, we suggest opening more Sejong Institutes in additional countries, deploying more excellent teaching staff, and offering a broader range of free Korean language courses. When students with at least a basic level of Korean language proficiency arrive in South Korea, they have better learning and living experiences, which will help enhance South Korea's reputation as a study-abroad destination and attract more international students. Thus, a mutually beneficial relationship can be established between the international promotion of the Korean language and the enhancement of South Korea's national image. Finally, international promotional policies for the Korean language should be formulated to match the characteristics of each country. Taking a developed country, the US, as an example, we suggest continuous efforts to promote the Korean Wave (Hallyu). Specific measures include organizing various cultural promotional activities related to Korean culture and providing policy support for the promotion of Korean TV dramas, movies, and music in the US market. In the case of Vietnam, a developing country, we recommend integrating the promotion of the Korean language with people's instrumental motivation to learn Korean. Specific measures involve providing Korean language learning resources that cater to the particular needs of learners based on their motivations, such as employment, studying abroad, and immigration.

This study analyzes the determinants of Korean language international promotion at a cross-country level, makes recommendations for the development of Korean language international promotion policies from an economic perspective, and may have the following implications or new insights: First, in terms of economics, we combine international Korean language promotion with economic factors for the first time. We propose the development of targeted Korean language promotion policies based on different countries' varying levels of economic development, which can contribute to the success of Korean language international promotion. Second, in terms of culture, our findings provide new insights by demonstrating the driving role of the Korean Wave in the internationalization of the Korean language for the first time at the national level. This study contributes to the existing research on the Korean Wave. Third, in Korean language education, we considered language-learning motivations at the national level for the first time. This offers a new perspective for research on Korean language education, especially when studying learners from multiple countries, by incorporating national-level factors into the analysis. Finally, this study has implications for the international promotion of other languages in other countries and can serve as a valuable reference.

Our study has several limitations. First, our theoretical component is relatively weak, as we only combined classical language learning and economic theories. Future research could use a deeper combination of cutting-edge language learning and economic theories to advance the development of both theories. Second, the lack of Korean language learner-level data makes it difficult to provide concrete guidance for practice in Korean language education. Therefore, we believe that a combined analysis of macro- and micro-level data in a subsequent study will contribute to Korean language education.

## Data availability statement

Data will be made available on request.

## CRediT authorship contribution statement

**Xingong Ding:** Writing – original draft, Visualization, Software, Methodology, Investigation, Formal analysis, Conceptualization. **Yujiao Wu:** Writing – review & editing, Writing – original draft, Validation, Supervision, Resources, Project administration, Funding acquisition, Data curation, Conceptualization.

## Declaration of competing interest

The authors declare that they have no known competing financial interests or personal relationships that could have appeared to influence the work reported in this paper.
